# Ultrasound-guided manual vacuum aspiration (USG-MVA) with cervical preparation for early pregnancy loss: A cost-effectiveness analysis

**DOI:** 10.1371/journal.pone.0294058

**Published:** 2023-11-03

**Authors:** Jacqueline Pui-Wah Chung, Ginenus Fekadu, Daljit Singh Sahota, Tak-Yeung Leung, Joyce H. S. You

**Affiliations:** 1 Department of Obstetrics and Gynaecology, Faculty of Medicine, The Chinese University of Hong Kong, Hong Kong, SAR, China; 2 School of Pharmacy, Faculty of Medicine, The Chinese University of Hong Kong, Hong Kong, SAR, China; National Academy of Medical Sciences, NEPAL

## Abstract

**Background and aim:**

Approximately one in four women will experience a miscarriage in their lifetime. Ultrasound-guided manual vacuum aspiration (USG-MVA) is an ideal outpatient surgical treatment alternative to traditional surgical evacuation. We aimed to examine the cost-effectiveness of US-MVA with cervical preparation for treatment of early pregnancy loss from the perspective of public healthcare provider of Hong Kong.

**Methods:**

A decision-analytic model was designed to simulate outcomes in a hypothetical cohort of patients with early pregnancy loss on four interventions: (1) US-MVA, (2) misoprostol, (3) surgical evacuation of uterus by dilation and curettage (surgical evacuation), and (4) expectant care. Model inputs were retrieved from published literature and public data. Model outcome measures were total direct medical cost and disutility-adjusted life-year (DALY). Base-case model results were examined by sensitivity analysis.

**Results:**

The expected DALYs (0.00141) and total direct medical cost (USD736) of US-MVA were the lowest of all interventions in base-case analysis, and US-MVA was the preferred cost-effective option. One-way sensitivity analysis showed that the misoprostol group became less costly than the US-MVA group if the evacuation rate of misoprostol (base-case value 0.832) exceeded 0.920. In probabilistic sensitivity analysis, At the willingness-to-pay (WTP) threshold of 49630 USD/DALY averted (1x gross domestic product per capita of Hong Kong), the US-MVA was cost-effective in 72.9% of the time.

**Conclusions:**

US-MVA appeared to be cost-saving and effective for treatment of early pregnancy loss from the perspective of public healthcare provider of Hong Kong.

## Introduction

Approximately 15%–20% of all clinically recognized pregnancies result in a miscarriage, and one in four women will experience a miscarriage in their lifetime [1]. Miscarriage can be managed through expectant, medical, or surgical methods. Expectant management involves waiting for the natural expulsion of the retained products of conception, but its success rate is often limited. Medical management typically employs a synthetic prostaglandin called misoprostol to expediate the process of evacuation of the products of conception.

Historically, surgical evacuation has been a common method for managing miscarriages. Surgical evacuation of the uterus by dilatation and curettage using sharp metal curettage is often performed in an operating theater under general anesthesia. However, surgical suction aspiration—either electronic or manual—has replaced sharp curettage due to its better safety profile and reduced complications. Cervical ripening is often performed to prepare the cervix, minimizing the risk of injury from cervical dilatation.

Manual vacuum aspiration (MVA) is a technique that utilizes a specially designed hand-held syringe with an attached silastic cannula for evacuation. Its effectiveness as a surgical alternative for the treatment of early pregnancy loss has been demonstrated. MVA offers several advantages, including its low cost, lightweight design, and portability. Additionally, it can be performed in an outpatient setting under local anesthesia without electricity. Despite no studies comparing the clinical outcomes of MVA with and without ultrasound guidance (USG), the integration of USG during the procedure ensures smooth introduction and correct placement of the catheter into the uterine cavity before applying suction, thus may reduce the rate of uterine perforation [2]. The introduction of USG-MVA has shifted the management of early pregnancy loss from the operating room to an ambulatory setting, allowing women to avoid the operating room and, in turn, reducing waiting times, admissions, and hospital stays, ultimately cutting healthcare costs.

To date, there is limited data available to evaluate the cost-effectiveness of USG-MVA with cervical preparation for the treatment of early pregnancy loss. The objective of this study was to evaluate the costs of expectant care, medical treatment with misoprostol, traditional surgical evacuation, and USG-MVA with cervical preparation for the treatment of early pregnancy loss.

## Materials and methods

### Model design

**Fig 1:**
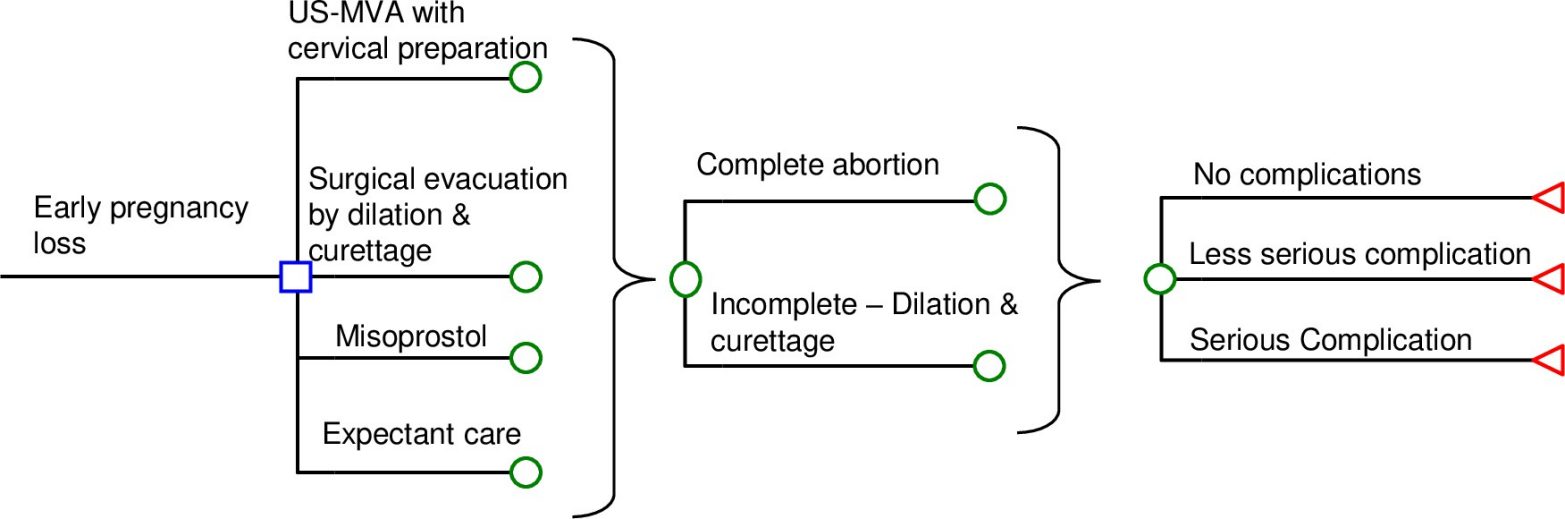
Simplified decision-analytic model.

A decision-analytic model (Fig 1) was designed to simulate outcomes in a hypothetical cohort of patients with early pregnancy loss (≤12 weeks of gestation) on four interventions: (1) USG-MVA with cervical preparation, (2) misoprostol, (3) surgical evacuation of the uterus, and (4) expectant care. The model timeframe was two weeks to allow adequate time for assessing the impact of all four interventions. Model outcome measures were total direct medical cost and disutility-adjusted life-year (DALY).

In the study arm of inpatient-based surgical evacuation, the hypothetical patients were hospitalized for one day for surgical evacuation, followed up by one clinic visit. The surgical evacuation might (or might not) achieve complete evacuation, and might (might not) cause complications. Incomplete evacuation was managed by repeated surgical evacuation. Complications included serious complications (blood transfusions, uterine perforations, hysterectomies, and intensive care unit admissions) and less serious complications (pelvic inflammatory disease, sepsis, and endometritis) [3].

In the arms of outpatient-based interventions (USG-MVA, misoprostol and expectant care), the patients were treated in the ambulatory setting. Each outpatient-based intervention might (or might not) achieve complete evacuation, and might (might not) cause complications. In-complete evacuation was managed by inpatient-based surgical evacuation.

### Clinical inputs

All model inputs are shown in **Table 1**. The clinical model inputs were retrieved from published literature. A MEDLINE search from the year 2000 to date was performed using keywords such as “early pregnancy loss”, “miscarriage”, “ultrasound-guided manual vacuum aspiration”, “misoprostol”, “dilation and curettage”, and “expectant care”. The selection criteria of clinical studies were: (1) Reports written in English, (2) patients with missed or incomplete miscarriage during the first 14 weeks of pregnancy, and (3) event rates of treatment effectiveness and/or complications were reported. Preferred studies were meta-analyses or randomized controlled trials. When multiple randomized trials were available for the same model input, the base-case value was estimated using the pooled average weighted against the number of patients in each study.

**Table 1:** Model inputs.

Parameter	Base-case value	Range for sensitivity analysis	Distribution	Reference
*Clinical inputs*				
Complete evacuation rate				
USG-MVA	100%	97.1%-100%	Beta	[3, 5]
Expectant care	64%	54%-74%	Beta	[3]
Misoprostol	83.2%	66.6%-99.8%	Beta	[3]
Surgical evacuation by dilation & curettage	95.4%	76.3%-100%	Beta	[3]
Less serious complication rate ^a^				
USG-MVA	0	0–2%	Beta	[3], assumption
Expectant care	3.6%	2.9%4.3%	Beta	[3]
Misoprostol	3.9%	3.1%-4.7%	Beta	[3]
Surgical evacuation	6.7%	5.4%-8.0%	Beta	[3]
Serious complication rate ^a^				
USG-MVA	0	0–2%	Beta	[3], assumption
Expectant care	1.9%	1.05%-2.75%	Beta	[3]
Misoprostol	1.0%	0.8% - 1.2%	Beta	[3]
Surgical evacuation	0.8%	0.6%-1.0%	Beta	[3]
*Utility inputs*				
Disutility–outpatient intervention	0.0367	0.0294–0.044	Triangular	[6]
Relative difference in disutility of dilation & curettage versus outpatient intervention	1.5	1.4–1.6	Triangular	Assumption
Relative difference in disutility of less serious complication versus outpatient intervention (no complication)	2	1.8–2.2	Triangular	Assumption
Relative difference in disutility of serious complication versus dilation & curettage (no complication)	2	1.8–2.2	Triangular	Assumption
*Cost inputs (USD)*				Local
USG-MVA	430	344–516	Gamma	
Misoprostol	2.6	0.85–3.91	Gamma	
Surgical evacuation	2224	1765–2681	Gamma	
Outpatient (per visit)	153	122–184	Gamma	
Number visit for outpatient intervention	2	1–3	Triangular	
Serious complication ^a^	5426	850–10004	Gamma	
Less serious complication ^a^	3230	850–10004	Gamma	
Hospitalization (per day)	654	523–785	Gamma	

^a^ Serious complications: Blood transfusions, uterine perforations, hysterectomies, and intensive care unit admissions; less serious complications: pelvic inflammatory disease, sepsis and endometritis.

A network meta-analysis on methods for managing miscarriage (including 78 randomized clinical trials (n = 17795)) had reported the comparative risks of the complete evacuation of expectant care, suction aspiration with cervical preparation, surgical evacuation, and misoprostol to be 640 per 1000, 1000 per 1000, 954 per 1000, and 832 per 1000, respectively. The comparative risks for serious complications and less serious complications of expectant care, surgical evacuation, and misoprostol were also reported [3]. The findings of comparative risks were adopted as the corresponding event rates in the present model. The network meta-analysis included one trial (n = 200) [4] for the suction aspiration with cervical preparation (100% complete evacuation) [3]. A local prospective cohort study of USG-MVA with cervical preparation (n = 35) also reported a high complete evacuation rate (97.1%) [5]. In the present model, the complete evacuation rate of USG-MVA with cervical preparation (by oral misoprostol) was approximated from the network meta-analysis and local cohort study (base-case 100%; range 97.1%-100%). The findings reported by the network meta-analysis and local cohort study both showed no serious or less serious complications with USG-MVA. The base-case values of complications, therefore, adopted zero value and assumed a range (0%-2%) to examine the impact of serious and less serious complications on the cost-effectiveness of USG-MVA.

### Utility and cost inputs

The DALYs of the hypothetical patients were estimated by the intervention-specific disutility and complication-specific disutility, in the two-week model period. A health economic analysis of outpatient-based intervention for treatment of early pregnancy loss reported that the health state (measured by Short Form Six Dimension) was reduced by 0.0367 over 2 weeks [6]. The present model adopted 0.0367 as the disutility value for the three outpatient-based interventions (USG-MVA with cervical preparation, expectant care and misoprostol). The relative difference in disutility between inpatient-based procedure (surgical evacuation) and outpatient interventions was assumed to be 1.5-fold. The disutility of less serious complication was assumed to be 2-fold of disutility of outpatient intervention. The disutility of serious complication was assumed to 2-fold of disutility of inpatient procedure (surgical evacuation).

The cost inputs were estimated from the perspective of the public healthcare provider in Hong Kong. The Hospital Authority is the largest public health organization in Hong Kong. The services provided by the Hospital Authority are subsidized by the government. Patients who are non-Hong Kong residents are billed by the charges of healthcare services posted in the Hong Kong Gazette. Assuming the charges listed in the Gazette represent only the cost components (including consumables, equipment maintenance, and staff costs) with no addition of profits, the costs associated with each treatment outcome were therefore approximated using the charges as listed in the Hong Kong Gazette.

### Cost-effective analysis and sensitivity analyses

The analysis was performed using TreeAge Pro 2021 (TreeAge Software Inc) and Excel 2016 (Microsoft Corporation). The expected cost and DALYs were generated in the base-case analysis. An intervention was the preferred cost-effective option if (1) it was the least costly with lowest DALYs, or (2) it gained the lowest DALYs at additional cost and the incremental cost per DALY averted (ICER = Δcost/ΔDALYs) was less than the willingness-to-pay (WTP). The World Health Organization recommended that ICER less than 1× gross domestic product (GDP) per capita to be highly cost-effective [7], and a threshold of 49630 USD/DALY averted (1x GDP per capita of Hong Kong [8]) was adopted as the WTP threshold.

The range for sensitivity analysis was the high/low values of the variable. If not available, a range of ± 20% of the base-case value was applied as the range for sensitivity analysis. One-way sensitivity analysis on all model inputs was performed to identify influential factors with threshold value. Probabilistic sensitivity analysis was performed by Monte Carlo simulations. Cost and DALY of each intervention arm were recalculated 10,000 times by simultaneously drawing all model inputs from the parameter-specific distribution (**Table 1**). The probability of each intervention to be accepted as cost-effective was examined over a range of WTP from zero to 150000 USD/DALY averted in the acceptability curves.

## Results

### Base-case analysis

The expected DALYs and total direct medical cost of USG-MVA were the lowest among all interventions (**Table 2**). Misoprostol, expected care and surgical evacuation resulted in higher DALY at higher cost than the USG-MVA, and were therefore dominated by USG-MVA. The USG-MVA was the preferred cost-effective option in the base-case analysis.

**Table 2:** Base-case results.

Strategy	Cost (USD)	Incremental cost (USD)	DALY	DALY averted
USG-MVA	$736	-	0.00141	-
Misoprostol	$1,009	$273	0.00193	-0.00052
Expectant care	$1,627	$891	0.00236	-0.00096
Surgical evacuation by dilation and curettage	$3,382	$2,646	0.00243	-0.00102

### Sensitivity analyses

**Fig 2:**
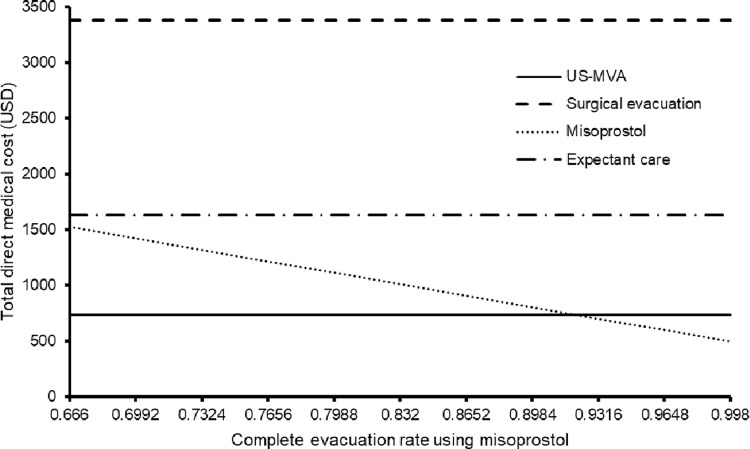
One-way sensitivity analysis of total direct cost versus complete evacuation rate of misoprostol.

The DALYs of the base-case results was robust and the USG-MVA remained the lowest throughout variation all model inputs in the one-way sensitivity analysis. The cost was sensitive to the complete evacuation rate of misoprostol. The misoprostol became less costly than the USG-MVA if the evacuation rate (base-case value 0.832) exceeded 0.920 (**Fig 2**).

**Fig 3:**
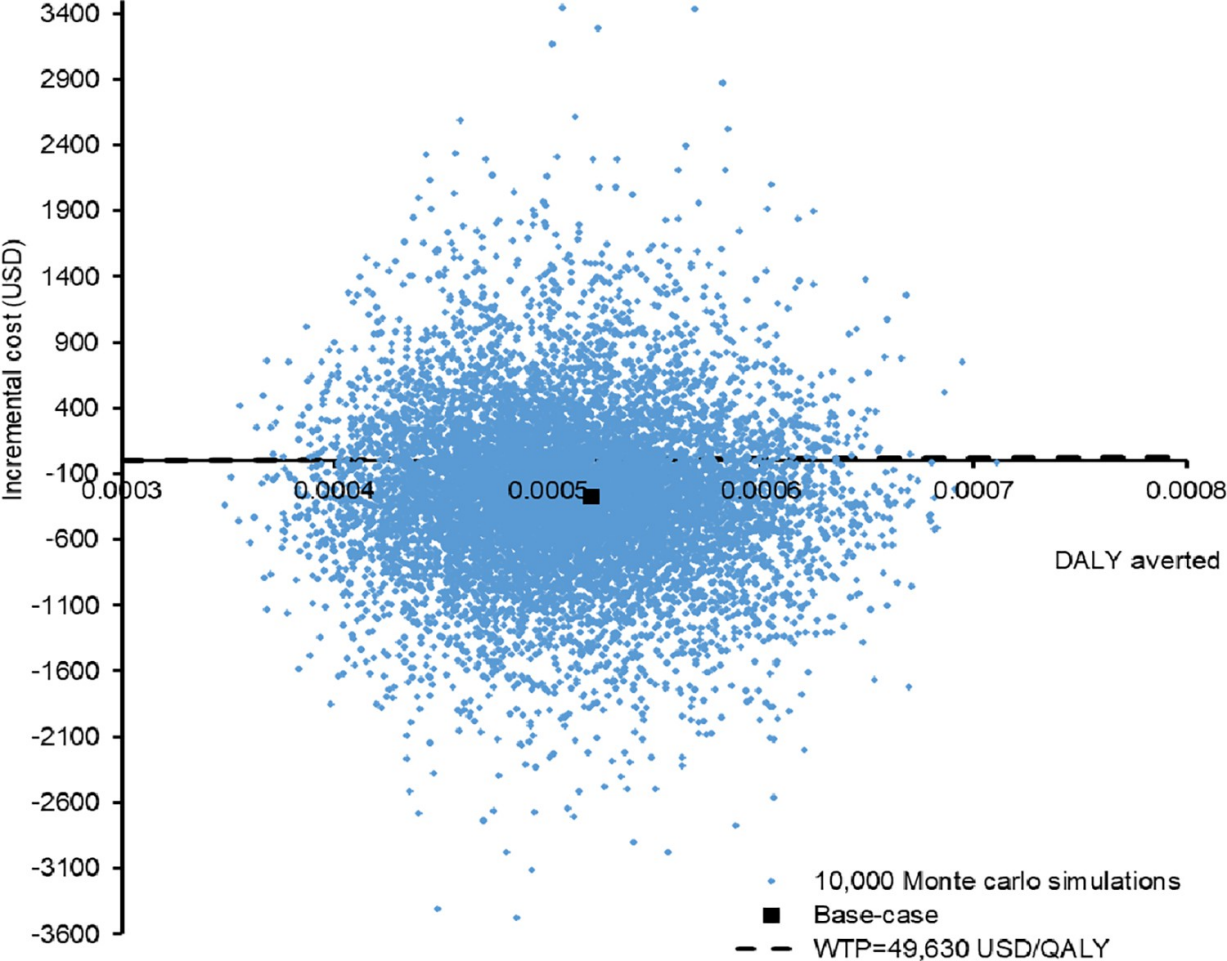
Scatter plot of incremental cost versus DALY averted by USG-MVA (comparing to misoprostol).

**Fig 4:**
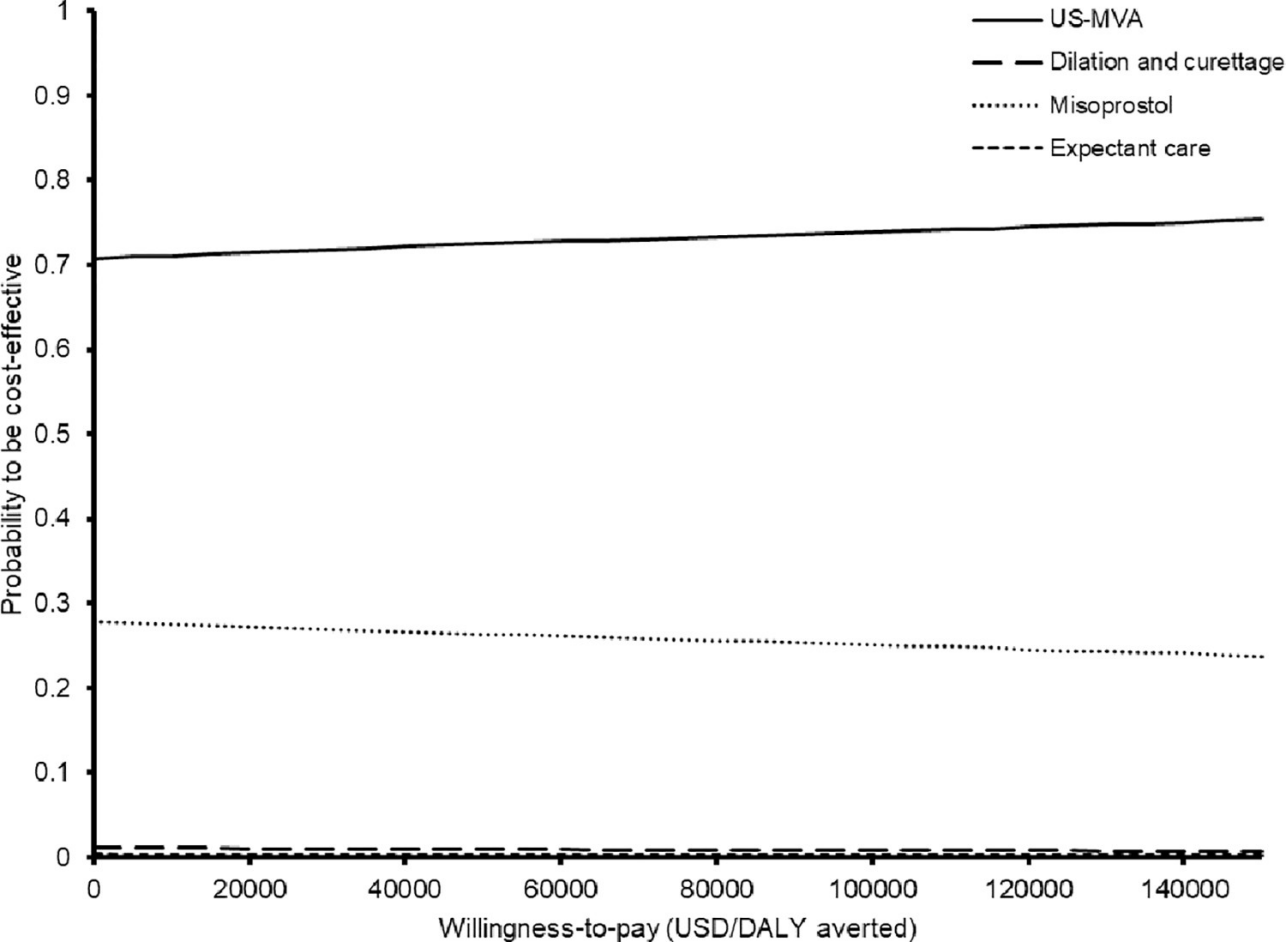
Acceptability curves of each intervention to be cost-effective against willingness-to-pay.

Probabilistic sensitivity analysis of the 10,000 Monte Carlo simulation was performed. The incremental cost versus DALY averted by USG-MVA (comparing to misoprostol) was showed in **Fig 3**. Comparing to misoprostol, the USG-MVA with cervical preparation was cost-saving by USD259 (95%CI 247–271; p<0.01), and averted 0.000509 DALYs (95%CI 0.000508–0.000510; p<0.01). At the willingness-to-pay (WTP) threshold of 49630 USD/DALY averted (1x GDP per capita of Hong Kong), the USG-MVA was cost-effective in 72.9% of the time. The probabilities of each intervention to be cost-effective against the variation of WTP threshold (from zero to 150000 USD/DALY) are showed in the acceptability curves (**Fig 4**). The probability of USG-MVA with cervical preparation to be cost-effective was the highest throughout the variation of WTP threshold.

## Discussion

Miscarriage is a significant healthcare issue worldwide, and there are four types of treatment options available for the management of early pregnancy loss, including expectant care, medical evacuation with misoprostol, USG-MVA, and surgical evacuation. However, limited health economic research has compared the cost-effectiveness of these four treatment options. In our previous cost analysis study on expectant, medical, and surgical treatment, misoprostol was found to be the least costly approach for the treatment of uncomplicated spontaneous miscarriage [9].

With the increase of patients in tertiary care hospitals and limited health and financial resources, procedures have moved from the operating room into the outpatient ambulatory setting. Our unit introduced USG-MVA in 2015, and we found it to be an effective, feasible, and safe surgical option for the management of early pregnancy loss in an outpatient setting under local anesthesia. The complete evacuation rate of USG-MVA was up to 97.1%, which is comparable to that of traditional suction evacuation (97.5%) reported in a previous systemic review [10]. USG-MVA can be easily performed in the outpatient setting with minimal complications and can result in substantial cost savings as it hastens the miscarriage process with low failure rate, and reduces the need for operation under general anesthesia. Therefore, we conducted further cost-effective analysis, comparing the cost and QALYs for all of four treatment options.

Our study findings confirm that USG-MVA is the preferred cost-effective option, with both the expected DALYs (0.00141) and total direct medical cost (USD736) being the lowest among all interventions in base-case analysis, and USG-MVA was the preferred cost-effective option. The complete evacuation rate of USG-MVA was the highest (100%) of the four interventions [2], and therefore reduced the needs for repeated surgical evacuation, and consequently lowered the total direct medical cost and DALY. The three other interventions’ complete evacuation rates were below 100%, resulting in the use of surgical evacuation for the in-complete evacuated cases, thus increased the cost and DALY. In the one-way sensitivity analysis, the misoprostol group became less costly than the USG-MVA group only if the evacuation rate of misoprostol (base-case value 0.832) exceeded 0.920. In probabilistic sensitivity analysis, at the willingness-to-pay (WTP) threshold of 49630 USD/DALY averted (1x gross domestic product per capita of Hong Kong), the USG-MVA was cost-effective in 72.9% of the time.

The findings of our study concur with previous literature that USG-MVA is a cost-effective surgical option. We found that the approach of using misoprostol would become less costly than USG-MVA if the complete evacuation rate of misoprostol increased from (base-case value) 83.2% to >92%, due to the reduced use of surgical evacuation for the in-complete evacuated cases (and therefore lowering the total direct medical cost). Misoprostol monotherapies have been extensively used for the medical management of early pregnancy loss. According to the largest randomized controlled trial conducted in the United States [11], complete expulsion rate was up to 71% by day 3 in women with first trimester pregnancy loss after one dose of 800 μg of vaginal misoprostol. The success rate was increased to 84% after a second dose of vaginal misoprostol if needed. The success rate of medical evacuation can be further increased if mifepristone is added as a combination therapy in the treatment of the early pregnancy loss [12]. However, mifepristone adds cost and may not be widely available in every unit, and may be limited to first- trimester medical induced abortions in some countries. In the United States, the availability of mifepristone is limited by the U.S. Food and Drug Administration Risk Evaluation and Mitigation Strategy restrictions [13].

Despite being simple, inexpensive, and easy to handle, the use of USG-MVA has been restricted as clinicians are not familiar with its use. We hope the findings that USG-MVA being more effective and less costly than misoprostol can increase the uptake of USG-MVA, and also change the clinical practice the treatment of early pregnancy loss, especially those suffering from recurrent miscarriages where cytogenetic analysis is also desired. Chorionic villi are the most direct and precious material for genetic investigation of the pregnancy loss. In our recent study, we confirmed the MVA approach was less traumatic and invasive than EVA in obtaining products of conception for analysis. The POCs derived from MVA are less disrupted, easier to be identified and also significantly reduces the culture failure rate of karyotyping and maternal cell contamination [14]. This additional advantage of USG-MVA can be taken in to reference in other healthcare systems with limited resources on while planning for healthcare management for early pregnancy loss. The cost-effectiveness analysis of the management options in low- or middle-income countries are highly warranted. The decision-analytical model developed in the present study provides a framework for the healthcare providers in other countries/regions. The model framework is readily applicable using country/region-specific clinical and cost parameters to examine the cost-effectiveness of outpatient USG-MVA (with cervical preparation) and other options for management of early pregnancy loss.

Previous studies have suggested that the use of USG-MVA may have a theoretical advantage in reducing the incidence of intrauterine adhesion (IUA) as it can minimize the damage to the endometrial lining as the use of USG can confirm the emptiness of the uterine cavity and avoid the need of further curettage [2]. IUA is known to be complication after surgical evacuation and can lead to negative impact in future fertility. It is also associated with reduction of the quality of life and is associated with significant healthcare economic consequences. Future studies are therefore warranted to evaluate the long-term cost-effectiveness of USG-MVA.

There are several limitations in our study. Model-based analyses are subject to uncertainty of model inputs. Most of the clinical model inputs were extracted from international clinical trials, the real time inputs may be different in outpatient settings, and therefore may affect the result generalization to patients in Hong Kong. Rigorous sensitivity analyses were therefore performed to examine the impact of model input uncertainty on the base-case results. A Hong Kong-specific WTP threshold has not yet determined, and a GDP-based WTP threshold was therefore adopted in the present study. We further found the USG-MVA with cervical preparation had the highest probability to be cost-effective throughout the variation of the WTP threshold. The model included expectant management, a less popular option in these days, yet still commonly offered to patients [15]. The medical management (with misoprostol) in the model was provided at ambulatory settings. Telemedicine has been widely accepted since COVID. Future health economic research on medical management via telemedicine is warranted. Also, the patient’s acceptance to different interventions was not considered in the present model. Further health economic study should therefore assess the impact of patient preference to various interventions. Our cost-analysis study only included direct medical costs. Indirect costs (productivity loss related to incomplete evacuation, such as absence from work and complications) were not considered in the analysis, and might therefore underestimated the health benefit generated by the high evacuation rate and low incidence of complications associated with USG-MVA (plus cervical preparation). Moreover, the time frame of our cost-analysis was only limited to two weeks. The economic impact of long-term complications including future fertility and psychological stress were not included in the present analysis. Future research to evaluate long-term real-world data of early pregnancy loss management in Hong Kong is warranted.

## Conclusions

In conclusion, our cost-effectiveness analysis study shows that outpatient USG-MVA with cervical preparation appears to be a cost-saving and effective treatment option for early pregnancy loss from the perspective of public healthcare provider of Hong Kong. Our results can be considered in other healthcare systems with limited resources.
